# ROR2 Promoter Methylation Change in Osteoblastic Differentiation of Mesenchymal Stem Cells

**Published:** 2011-04-21

**Authors:** Gorbanali Tarfiei, Mehrdad Noruzinia, Masood Soleimani, Saeed Kaviani, Maryam Mahmoodinia Maymand, Majid Farshdousti Hagh, Pascal Pujol

**Affiliations:** 1. Department of Hematology, Tarbiat Modares University, Tehran, Iran; 2. Laboratory of Epigenetics in Human Diseases and Stem Cells, Department of Medical Genetics, Tarbiat Modares University, Tehran, Iran; 3. Stem Cell Laboratory, Sarem Cell Research Center (SCRC), Tehran, Iran; 4. Service de Genetique Medicale, Inserm 896, CHU de Arnaud-de-Villeneuve, 371, Avenue du Doyen-Gaston-Giraud, 3295 Montpellier Cedex, France

**Keywords:** Mesenchymal Stem Cell, Methylation, ROR2, Osteoblast Differentiation, Epigenetics

## Abstract

**Objective:**

Osteoblasts arise from multipotent mesenchymal stem cells (MSCs) present in the bone marrow stroma and undergo further differentiation to osteocytes or bone cells. Many factors such as proteins present in the Wnt signaling pathway affect osteoblast differentiation. ROR2 is an orphan tyrosine kinase receptor that acts as a co-receptor in the non-canonical Wnt signaling pathway. However, ROR2 has been shown to be regulated by both canonical and non-canonical Wnt signaling pathways. ROR2 expression increases during differentiation of MSCs to osteoblasts and then decreases as cells differentiate to osteocytes. On the other hand, research has shown that ROR2 changes MSC fate towards osteoblasts by inducing osteogenic transcription factor OSTERIX. Here we speculated whether ROR2 gene expression regulation during osteoblastogenesis is epigenetically determined.

**Materials and Methods:**

MSCs from bone marrow were isolated, expanded and characterized *in vitro* according to standard procedures. ROR2 promoter methylation status was determined using methylation specific PCR in a multipotent state and during differentiation to osteoblasts.

**Results:**

We determined that the demethylation process in ROR2 promoter occurs during the differentiation process. The process of demethylation begins at day 8 and continues until 21 days of differentiation.

**Conclusion:**

This result is in concordance with previous works on the role of ROR2 on osteoblast differentiation, which have shown an upregulation of ROR2 expression during this process.

## Introduction

Osteoblasts are derived from mesenchymal stem cells (MSCs) residing in the bone marrow stroma. These cells undergo further differentiation to osteocytes when necessary (1, 2). The transition from stem cells to mature osteoblasts is characterized by the formation of a mineralized extracellular matrix (1). A large variety of hormones, growth factors, cytokines and vitamins are involved in osteoblast differentiation (1). Transcription factors such as runt-related transcription factor 2 (RUNX2), osterix (OSX) and distal-less homeobox 5 (DLX5) are key regulators of the differentiation process. These factors are activated via different signaling pathways (1, 3). On the other hand it has been shown that the Wnt signaling pathways (either canonical or non-canonical) particularly play an important role in bone formation (4, 5).

Wnt molecules are secreted, lipid-modified glycoproteins structurally related that share more than 20 cysteine residues. Wnts bind to serpentine receptors of the frizzled (FZD) family on the plasma membrane to initiate several distinct cascades classified as either canonical or non-canonical, depending on whether β-catenin is involved. So far, at least 19 Wnt proteins have been identified in mammals (5). Classically, Wnt1, 2, 3 and 3a, 8 and 8b are regarded as the canonical Wnts; Wnt4, 5a, 5b, 6, 7a and 11 are non-canonical (5). Wnt proteins activate cell surface receptor-mediated signal transduction pathways hence regulating a variety of cellular activities, including cell fate determination, proliferation, migration, polarity and gene expression (6, 7). Recent studies have demonstrated that both the canonical and non-canonical cascades control lineage specification and the early differentiation potentials of human MSCs. In the process of MSC osteogenesis, Wnt11, FZD6, sFRP2, sFRP3 and ROR2 are up-regulated while Wnt9a and FZD7 are down-regulated. Canonical Wnt signaling appears to suppress osteogenic differentiation. However, the effect of canonical Wnt signaling on MSC osteogenesis may vary according to the level of Wnt activity. On the other hand, the effect of canonical Wnt signaling on osteogenesis also depends on the stage of the target cells. Once MSCs are committed to osteogenic lineage, canonical Wnt signaling stimulates their differentiation, while at the same time inhibiting the terminal differentiation of mature osteoblasts.

ROR2 (HGNC: M97639) belongs to a family of orphan receptor tyrosine kinases (8). In mammals this family consists of two members, ROR1 and ROR2. ROR2 plays crucial roles in developmental morphogenesis, particularly of the skeleton. Expression of ROR2 mRNA is highly regulated in a biphasic manner during human osteoblast differentiation. While virtually undetectable in pluripotent stem cells, its expression is increased 300-fold in committed pre-osteoblasts and disappears again in osteocytes. Furthermore, Wnt antagonist secreted frizzled-related protein 1 can suppress ROR2 expression in osteoblasts. These evidences suggest that ROR2 may regulate bone formation (1, 9, 10). Gene expression in undifferentiated cells can be regulated by epigenetic processes at DNA and chromatin levels situated at or near gene regulatory and coding regions. A well characterized epigenetic modification is cytosine methylation, which is generally associated with long-term gene silencing. DNA methylation consists of the addition of a methyl group to the 5 position of a cytosine in a cytosine–phosphate–guanine (CpG) dinucleotide (11). This addition is catalyzed through an enzymatic reaction which uses S-adenosyl-methionine as a methyl group donor and action of DNA methyltransferases (DNMT) (12). CpG methylation is symmetrical and targets isolated CpGs, clustered CpGs, or even CpGs within a CpG island. A CpG island is defined as either (i) a 200 bp window moving across a sequence of interest at 1 bp intervals, with a C+G content > 50% and an observed/expected CpG frequency of >0.6, or (ii) a 500 bp moving window with a C+G content of >55% and an observed/ expected CpG frequency of >0.65 (11).

CpG islands are usually found in the 5′ regulatory regions of vertebrate housekeeping genes (11). Promoters with low CpG content show no correlation between gene expression and abundance of methylated CpGs; therefore, transcriptionally active low CpG promoters (LCPs) are not necessarily un- or hypomethylated. It seems in fact that most low CpG promoters are methylated regardless of their activity status. On the contrary, the activity of intermediate CpG content promoters (ICPs) and high CpG content promoters (HCPs) is inversely correlated to the extent of methylation. In these categories, the proportion of transcriptionally active promoters decreases with increasing DNA methylation, arguing that methylation of ICPs and HCPs is incompatible with transcription (13).

Aranda et al. have shown that the differentiation potential in stem cells is associated with epigenetic status (14). Indeed, epigenetic modifications at several genes, such as methylation of the promoter region and the subsequent down-regulation of gene expression, have been shown to play an important role in cell differentiation. We decided thus to study methylation status of the ROR2 promoter region during *in vitro* MSC to osteoblast differentiation.

## Materials and Methods

### Isolation and culture of hBMSCs

Bone marrow aspirate was obtained from the iliac crest of a human healthy donor at the Bone Marrow Transplantation Center, Shariati Hospital, Tehran, Iran. The donor gave informed consent and the Ethical Committee of Tarbiat Modares University approved the study.

Briefly, the aspirate was diluted with Hank’s balanced salt solution (HBSS) without calcium or magnesium. The cell solution was gently overlaid on a Ficoll gradient to separate unwanted cell types present in the marrow aspirate. The mononuclear cell layer at the interface of the Ficoll and HBSS were collected after centrifugation at 1800 g for 30 minutes at room temperature. Isolated mononuclear cell layers were re-suspended in HBSS and centrifuged at 1000 g for 10 minutes at room temperature followed by a repeat of the washing procedure. The cell pellet was re-suspended in growth medium containing DMEM-low glucose supplemented with 15% (v/v) FBS, 2-mM glutamine, 100 µg/ml streptomycin, 100 U/ml penicillin, and plated in 75 cm^2^ polystyrene plastic cell culture flasks (15). The cell culture flasks were incubated overnight at 37℃ in a humidified incubator under 5% CO_2_ and then non-adherent cells were removed, leaving behind the adherent cell population: washings with phosphate buffered saline without calcium or magnesium (PBSA) and medium replenishment were repeated every second day for six days. When the adherent, spindle-shaped fibroblastoid cells reached 50-60% confluency, cells were harvested with 0.25% (w/v) trypsin-EDTA solution and plated in 25 cm^2^ cell culture flasks at a density of 104 cells/cm (15).

### Flow cytometric analysis of hBMSCs

Flow cytometry was performed at the Iranian Blood Transfusion Organization. hBMSCs were detached from the cell culture flasks after 12 days (second passage) with a trypsin-EDTA solution and washed with PBSA. The cells were re-suspended in PBSA and counted. About 1×10^6^ cells were divided into aliquots and centrifuged at 1000 rpm for 5 minutes at room temperature. The cell pellet was resuspended in human serum and incubated for 30 minutes on ice. After centrifugation at 1000 rpm for 5 minutes, the pellet was re-suspended in 3% (v/v) human albumin serum (HAS)/PBS and incubated with appropriate antibodies that included fluorescent isothiocyanate (FITC) conjugated anti- human CD44, CD13, CD34 and phycoerythrin (PE) conjugated anti-human CD45, CD166 and CD105 for 1 hour on ice, washed twice in PBS and centrifuged for 5 minutes. Cells were re-suspended in 100 µl of PBS and analyzed with a Partec PAS III flow cytometer. The negative control was an isotype control with FITC or PE labeled IgG1.

### Osteoblast differentiation

For osteoblastic differentiation, hBMSCs were cultured at 37℃ in a humidified incubator under 5% CO_2_ for 21 days by bone differentiating medium (BDM) containing α-MEM supplemented with 10% (v/v) FBS, 2 mM glutamine, 100 µg/ml streptomycin, 100 U/ml penicillin, 5 mM β-glycerol phosphate, 50 µg/ml ascorbate-2-phosphate and 10 nM dexamethasone in T25 culture flasks and sixwell plates. BDM was changed each three days. Differentiating cells on days 4, 8, 12, 16 and 20 were harvested from culture flasks with the use of a trypsin-EDTA solution and DNA or RNA were extracted.

The six-well plates were used for alizarin red staining (ARS).

### Alizarin red staining

At day 21, the differentiated cells in the six-well plates were washed twice with PBSA and fixed by formalin at room temperature for 10 min. Formalin was removed from the wells and the cells were washed twice with PBSA and once with distilled water. Then, ARS solution was added to the wells and plates were incubated at room temperature for 30 minutes. Finally the wells were washed with distilled water until the background staining on the ‘‘negative’’ wells (wells containing MSCs) was maximally cleared. Cells were examined by an invert microscope.

### DNA extraction

DNA Extraction Kit (Roche, cat. no: 11796828001) was used to extraction DNA from MSCs and osteoblastic cells according to the manufacturer’s insctructions. Briefly, MSCs were harvested with a trypsin-EDTA solution and washed twice with PBS. Then, the cell pellet was re-suspended in 200 µl PBS and transferred into a 1.5 microtube. After adding 200 µl binding buffer and 40 µl proteinase K, the solution was mixed immediately and incubated at 70℃ for 10 minutes. After incubation, 100 µl isopropanol was added and mixed well.

A filter tube was inserted in a collection tube and the samples were transferred into the filter tube. After centrifugation for 1 minute at 8000 g, the filter tube was removed from the collection tube and combined with another collection tube. Then, 500 µl of inhibitor buffer was added to the filter tube and centrifuged for 1 minute at 8000 g. The washing procedure was repeated twice. Finally, the filter tube was inserted in a sterile 1.5 ml microtube and 200 µl prewarmed elution buffer was added into the filter tube and then centrifuged for 1 minute at 8000 g. The microtube contained eluted DNA.

### RNA extraction

RNA extraction from MSCs and osteoblasts at day 21 was performed using the RNA Extraction Kit (Rima zol) according to the manufacturer’s instructions. Briefly, MSCs were lysed directly in the culture flasks by adding 2.5 ml of Rima zol into T25 tissue culture flasks (1 ml per 10 cm^2^ of culture dish area) and passing the cell lysate through a pipette several times. After transferring the cell lysate into a 15 ml tube, 500 µl of chloroform was added and shaken vigorously for 15 seconds. Then the mixture was incubated on ice for 5 minutes and centrifuged at 12000 rpm for 15 minutes at 4℃. Following centrifugation, the mixture separated into a lower phase, an interphase and a colorless upper aqueous phase. The aqueous phase containing RNA was transferred to a 1.5 ml microtube and mixed with an equal volume of isopropanol. The mixture was incubated at -20℃ for 10 minutes and then centrifuged at 12000 rpm for 10 minutes at 4℃. After the supernatant was removed, 1 ml of 80% ethanol was added to the RNA pellet, mixed well by vortexing and centrifuged at 12000 rpm for 5 minutes at 4℃. Finally, the supernatant was removed and the RNA pellet dissolved in RNase-free water by pipetting and incubating for 10 minutes at 55 to 60℃.

### Reverse transcription polymerase chain reaction (RT-PCR)

RT-PCR was used to determine the expression of osteoblastic cell markers, alkaline phosphatase (ALP) and osteocalcin (OSC). cDNA synthesis and the PCR reaction were performed as described below.

### RT reaction

Total RNA at an amount of 10 µl (5 µg) was incubated at 65℃ for 10 minutes after which the following components were added: 3 µl of 10X PCR buffer, 2.5 µl dNTP (10 mM), 6 µl MgCl_2_ (25 mM), 1 µl of random primer, 0.5 µl reverse transcriptase and 17 µl DDW.

After incubation at 25℃ for 10 minutes, the samples were incubated at 42℃ for 1 hour.

### RT-PCR for alkaline phosphatase

ALP is an early marker of osteoblastic cells. In this assay, the PCR reaction was performed by using osteoblastic cDNA and MSCs cDNA to determine OSC expression in the osteoblastic cells as compared with MSCs. Primers are shown in table 1.

The PCR mixture contained ×l0 PCR buffer, Mg- Cl_2_ (1 mM), dNTP (each at 200 µM), primers (0.1 mM each primer), cDNA (100 ng) and Taq DNA polymerase 2 U per reaction in a final volume of 25 µl.

After initial denaturation at 94℃ for 3 minutes, PCR amplification continued at the following: a) 94℃ for 30 seconds and b) an annealing temperature of 58℃ for 20 seconds and 72℃ for 30 seconds for a total of 35 cycles, followed by a final extension at 72℃ for 7 minutes. The amplified DNA fragments were electrophoresed on a 1.5% (w/v) agarose gel. The gel was stained with ethidium bromide (10 µg/ ml) and photographed on a UV transilluminator (Bio Doc).

### RT-PCR forosteocalcin

OSC is a late marker of osteoblastic cells. As with the ALP assay, the PCR reaction was performed by using osteoblastic cDNA and MSCs cDNA to determine OSC expression in the osteoblastic cells as compared with MSCs. Primers are shown in Table 1.

The PCR mixture contained: ×l0 PCR buffer, Mg- Cl_2_ (1.5 mM), dNTP (each at 200 µM), primers (0.1 mM each primer), cDNA (100 ng) and Taq DNA polymerase 2 U per each reaction in a final volume of 25 µl.

An initial denaturation at 94℃ for 3 minutes was followed by 35 cycles of denaturation step at 94℃ for 40 seconds with an annealing temperature at 64℃ for 20 s and extension step at 72℃ for 30 seconds, with a final extension at 72℃ for 5 minutes.

Amplified fragments were separated by electrophoresis on a 1.5% (w/v) agarose gel. The gel were stained with ethidium bromide (10 µg/ml).

### Methylation-specific PCR (MSP)

#### Bisulfite modification

Briefly, 10 µl DNA from PB, MSCs and osteoblastic cells were denatured with 0.2 mol/l NaOH for 10 mintes at 37℃ in 50 ml total volume. About 30 ml of 10 mmol/l hydroquinone and 520 ml of 3.5 mol/l sodium bisulfite (pH=5.0) were added. DNA samples were incubated under mineral oil at 50℃ for 16 hours. Samples were then purified using Qiagen DNA purification columns. Recovered samples were desulfonated in 0.3 M NaOH for 5 minutes at room temperature. After ethanol precipitation, DNA was dissolved in 40 ml water and used immediately for PCR amplification or stored at -20℃.

#### PCR reaction with methylated primers

MSP was performed in a total volume of 25 ml. PCR reactions for both primer sets contained 12.65 µl DDW, 2 mM MgCl_2_, 0.2 mM dNTP, 2.5 µl 10X PCR buffer, 3 µl (100 ng) DNA, 0.8 µM of each primer and 2 U Taq DNA polymerase (Cinnagen, Iran). An initial denaturing step at 95℃ for 5 minutes was followed by 35 cycles at 95℃ for 40 seconds, 56℃ for methylated primers (50℃ for unmethylated primers) for 40 seconds and 72℃ for 35 seconds and a final extension at 72℃ for 10 minutes.

One control PB DNA sample was methylated using Sss1 methyltransferase (New England BioLabs) according to the manufacturer’s protocol and used as a methylated, positive control for MSP reactions.

## Results

After MSC differentiation and confirmation by flow cytometry, all steps were repeated in triplicate to ensure reproducibility.

### Characterization of hBMSCs

**Fig 1:**
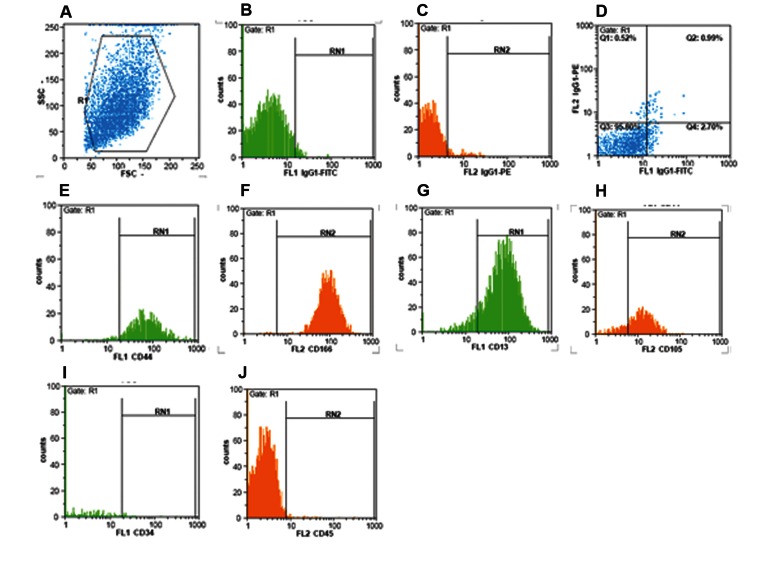
Flow cytometric analysis of hBMSCs. Flow cytometric analysis was performed for the specific markers of MSCs and hematopoietic markers. MSCs were positive for CD44, CD166, CD13 and CD105. These cells were negative for CD34 and CD45. A: MSCs size and granularity. B,C,D: Isotype control. E: CD44. F: CD166. G: CD13. H: CD105. I: CD34. J: CD45.

**Fig 2:**
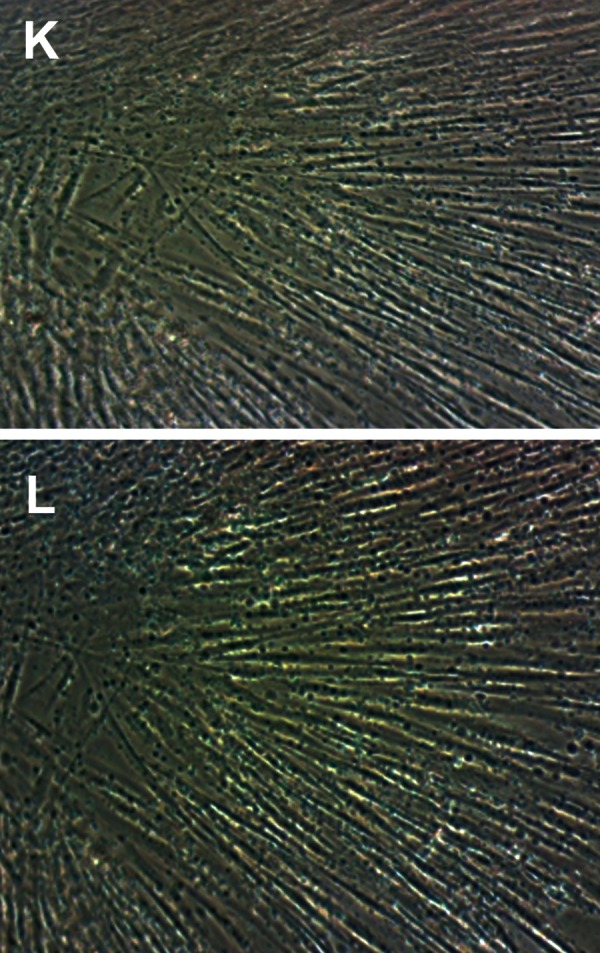
MSCs morphology by inverted microscope. K: First passage cells. L: Second passage cells. MSC: mesenchymal stem cells.

hBMSCs were cultivated from the mononuclear cell fraction of the human bone marrow sample obtained from a healthy donor. To ensure the removal of contaminating hematopoietic cells, the cells were selected based on plastic adherence and passaged twice prior to future use. To confirm that culture-expanded cells were genuine MSCs, their phenotype was examined. Flow cytometric analysis of these cells revealed expression of CD13, CD44, CD105 and CD166, but not CD34 and CD45 (Fig 1). Morphological observation of culture-expanded cells by an invert microscope exhibited small, spindle-shaped cells with refractile doublets (Fig 2). In summary, these results indicated that the expanded cells had the basic properties of MSCs.

**Fig 3:**
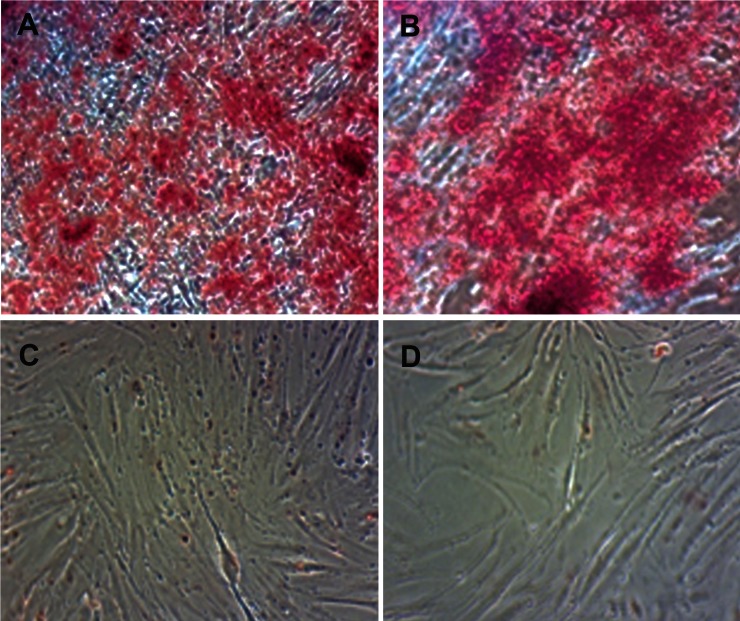
Alizarin red staining. A and B: Mesenchymal stem cells differentiated to osteoblasts on day 21. C and D: MSCs day 0 of differentiation (negative control).

### Osteoblast differentiation confirmation

#### ARS

ARS confirmed the presence of calcium deposits, characteristic of osteogenic cells, whereas undifferentiated MSCs were negative for ARS (Fig 3).

#### Osteoblast specific gene expression

**Fig 4:**
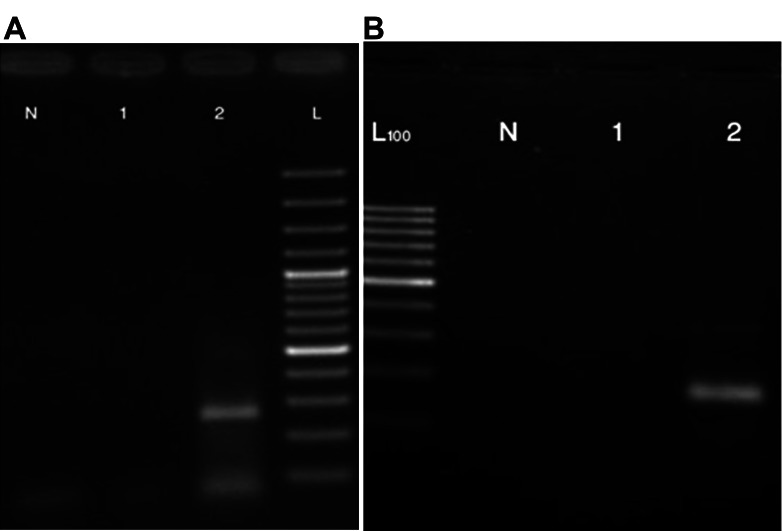
RT-PCR for osteoblast specific gene. A: Osteocalcin marker expressed in the osteoblasts (No. 2) but not in MSCs (No. 1). N: Negative control. B: Alp marker expressed in the osteoblasts (No. 2) but not in MSCs (No. 1). N: Negative control. L: Ladder 100 bp.

Our findings confirmed osteoblastic cell generation by RT-PCR for the well known markers of osteoblast differentiation such as ALP and osteocalcin. As shown in fig 4, undifferentiated MSCs did not express mRNA of the osteoblast lineage genes but differentiated osteoblastic cells expressed the studied genes

#### Methylation-specific PCR

**Fig 5:**
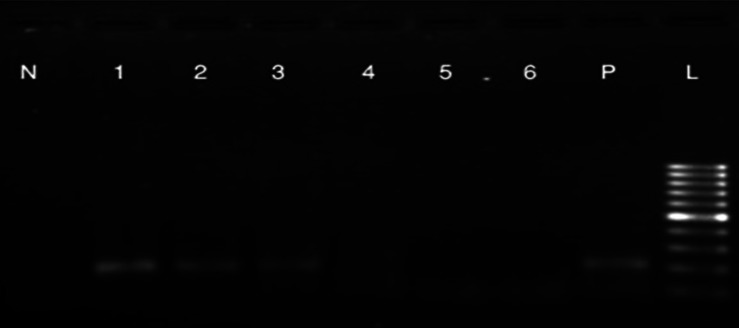
MSP results (with methylated DNA specific primers). N: negative control. 1: MSCs. 2: osteoblast on day four. 3:osteoblast on day eight. 4: osteoblast on day twelve. 5: osteoblast on day sixteen. 6: osteoblast on day twentieth. P: positive control. L: Ladder 100bp. Product size: 216bp.

MSP results indicated that the 5' end of the ROR2 gene in MSCs is methylated, but unmethylated in osteoblastic cells. As shown in fig 5, MSP with methylated primers amplified the promoter region of the ROR2 gene in MSCs and osteoblastic cells on days 4 and 8 of differentiation, but not in the osteoblastic cells on days 12, 16 and 20.

**Fig 6:**
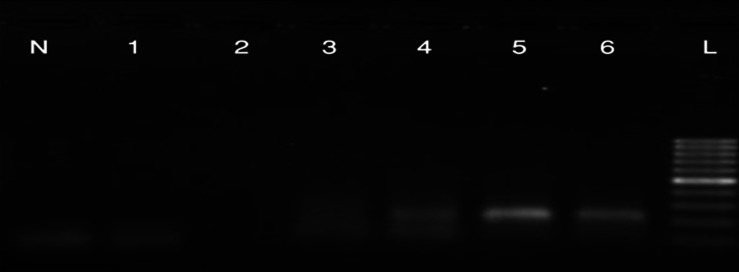
MSP results (with un-methylated DNA specific primers). N: negative control. 1: MSCs. 2: osteoblast on day four. 3:osteoblast on day eight. 4: osteoblast on day twelve. 5: osteoblast on day sixteen. 6: osteoblast on day twentieth. L: Ladder 100bp. Product size: 219bp.

Contrary to MSP with methylated primers, MSP with un-methylated primers amplify the promoter region on days 12, 16 and 20 of differentiation, while producing no PCR product on days 4 and 8 of differentiation.(Fig 6)

These findings suggest that the promoter region of the ROR2 gene is methylated in the MSCs and osteoblastic cells until day 8 after which it becomes hypo-methylated. These results support previous findings which showed an increase in expression of ROR2 mRNA during osteoblastogenesis.

## Discussion

ROR2 has been shown to be regulated by both canonical and non-canonical Wnt signaling pathways. On the other hand, expression of ROR2 mRNA was shown to be regulated during osteoblast differentiation which suggested that ROR2 may regulate bone formation (1, 9). In addition Liu et al. have demonstrated that ROR2 initiates osteoblastic lineage commitment of human mesenchymal stem cells (hMSC) that have the ability to differentiate into several distinct lineages (16).

Knowledge of the mechanism of ROR2 gene expression regulation is important and would help to decorticade the pathogenesis of skeletal dysplasias with osteoblastic differentiation. Theoretically, regulation of ROR2 expression could be influenced by different mechanisms. It has been shown that in stem cell differentiation, DNA sequencespecific transcription factors can be involved in many genes.

DNA methylation is one of the main mechanisms of transcription methylation in CpG rich promoters. ie. 50% of tissue specific genes and the majority of housekeeping genes (17). In this research we show that ROR2 gene expression is regulated by methylation change of it's promoter.

Epigenetic regulations are very complex and many interacting pathways are involved. It has been shown that epigenetic regulation could be influenced at different levels. Methylation of DNA is one of the most frequent epigenetic mechanisms. However promoter methyltion is not always associated with transcriptional repression. This depends upon the CpG content of promoters. Methylated high CpG promoters are usually inactive and methylated low CpG promoters can be active or inactive (18).

We show in concordance to previous studies on mRNA that the ROR2 promoter is progressively methylated during differentiation. It has interestingly been shown in our previous work and many others that methylation is mostly tissue and gene specific (19).

Our result is important as it has been shown previously that in stem cells, gene expression is mostly regulated at the chromatin level. It has been shown that most important genes in stem cells have a bivalent state. Active and repressive chromatin state (H3K4 methylation and H3K27 methylation, respectively) is a characteristic of a bivalent state. DNA methylation of CpG rich sequences is very low and limited in stem cells (20, 21).

A large body of evidence in the past few years has indicated that chromatin could influence maintenance and regulation of transcriptional programs (17). We have shown in this study that DNA methylation is not excluded as a regulating mechanism in stem cell differentiation.

Our result is supported by previous studies which show that some promoters of stem cells are one of the main targets of de novo DNA methylation (21, 22). For example, Hayashi et al. have shown that stella is partially methylated in embryonic stem cells (23). DNA methylation can shape mammalian promoter structure and stabilize pluripotency shut-down during differentiation.

Taken together, stem cell to progenitor differentiation is not associated with extensive DNA methylation changes (24, 25). ROR2 methylation change in its promoter region is likely to be more specific than due to global methylation changes. Sorensen et al. have proposed that the promoter methylation state in progenitor cells constitutes a ground state program of gene activation potential with strong methylation being repressive and hypomethylation being potentially permissive (26). In fact, DNA methylation plays a crucial role in fixation of cell lineage fate in differentiated embryonic stem cells (27).

We have shown that the demethylation process begins at the ROR2 promoter about eight days of differentiation. This finding is in concordance with previous finding on ROR2 expression showing that ROR2 is not expressed in MSCs. ROR2 is upregulated during osteoblast differentiation, being the highest in the pre-osteoblast stage (1).

## Conclusion

Our results, in addition to the results of others, show that ROR2 modulates MSC to osteoblast differentiation through at least methylation dependent gene expression regulation at around eight days of differentiation.

This is the first report on epigenetic regulation of the ROR2 gene in osteoblastic differentiation. We found that methylation is involved in expression modification of ROR2. Methylation in the CpG island has been proven to cause down regulation of gene expression. Our results are in concordance with previous findings that during osteoblast differentiation ROR2 expression changes. We have confirmed that this change in expression occurs through CpG island demethylation during osteogenesis.
